# Global research trends in acupuncture for cancer pain: A bibliometric analysis

**DOI:** 10.1097/MD.0000000000034739

**Published:** 2023-10-13

**Authors:** Xia Yang, Bing Liang, Demin Xue, Jing Liang, Chris Zaslawski, Ji Chen

**Affiliations:** a Basic Medical College, Chengdu University of Traditional Chinese Medicine, Chengdu, China; b School of Chinese Classics, Chengdu University of Traditional Chinese Medicine, Chengdu, China; c School of Foreign Languages, Chengdu University of Traditional Chinese Medicine, Chengdu, China; d Faculty of Science, University of Technology, Sydney, New South Wales, Australia.

**Keywords:** acupuncture, bibliometric analysis, cancer pain, CiteSpace, visualization, VOSviewer

## Abstract

**Background:**

In recent years, acupuncture has gained popularity in the management of cancer-related pain (CRP). This study aims to use bibliometric analysis to investigate the historical development, recent hotspots and research trends in this field.

**Methods:**

The Web of Science Core Collection database was selected as the data source for this study to retrieve and obtain literature related to acupuncture and CRP. Data analyses were performed with CiteSpace and VOSviewer to conduct the bibliometric analysis.

**Results:**

This bibliometric analysis was conducted from 2000 to 2022. A total of 664 publications were included in this work. The number of publications has steadily increased over the last 2 decades. The United States has the largest number of published articles (244 papers), while the People’s Republic of China has the highest centrality (0.48). The primary research institutions were Memorial Sloan-Kettering Cancer Center, Kyung Hee University and Beijing University of Chinese Medicine. Mao Jun J. was the most prolific author, while Heather Greenlee was the most cited one. The most productive journal was Integrative Cancer Therapies. The most frequent keywords excluding the search subject were “electroacupuncture,” “management,” “quality of life,” “breast cancer,” “Aromatase inhibitor,” “neuropathic pain,” “mechanisms,” and “protocol.”

**Conclusion:**

This study explored the application value of acupuncture in the management of CRP with bibliometric analysis, offering an intuitive understanding of this topic and revealing the hotspots and research trends.

## 1. Introduction

Cancer has become a major public health problem worldwide, with more than 17 million cases reported worldwide each year (Cancer Research UK).^[1]^ It markedly increases the economic and social burden for both individuals and the community^[2,3]^ According to overall cancer pain prevalence data, the presence of pain at the time of cancer diagnosis was 35%, and the average prevalence of moderate to severe pain throughout the course of the disease was 46%, rising to 76% in patients with advanced cancer.^[4]^ Pain is one of the most common and distressing symptoms for cancer patients, which seriously affects their quality of life. At the same time, there are many side effects of opioids when used for cancer pain, such as constipation, nausea, vomiting and moral condemnation,^[5,6]^ which undoubtedly increases the physical and mental burden for patients. There is growing evidence that acupuncture is being recognized in the National Comprehensive Cancer Network guidelines as one of the complementary and alternative therapies for the treatment of cancer pain and improving function due to its low side effects.^[7,8]^ Acupuncture has been practiced in China for more than 2000 years, and it is a physical intervention which involves insertion fine needles at different acupoints in the skin. Acupuncture is widely used in China for its efficacy and low side effects, and in recent years it has been gradually accepted in other countries as well. Many studies have been conducted to investigate the use of acupuncture in the treatment of pain and shown acupuncture was commonly used for many types of chronic pain,^[9]^ electroacupuncture (EA) could improve neuropathic pain by reducing P2X7R and P2X7R,^[10]^ EA could improve the pain threshold in rats with Neuropathic pain.^[11]^

Bibliometrics is a quantitative tool for published scholarly literature based on mathematical and statistical analysis. It is used to measure interrelationships, the impact of the publications, and trends in a given field of study. Bibliometric network analysis, co-occurrence analysis, co-authorship and co-citation are used to create visual graphs that summarize the network relationships in the field of study. The use of acupuncture for pain, low back pain, and stroke rehabilitation has become popular worldwide^[12–14]^ and the number of studies contributing to acupuncture in the field of cancer-related pain (CRP) has also increased annually over the past decades. However, there are few reports on the overall trends of acupuncture for the management of CRP based on bibliometric analysis.

The aim of this study was to systematically provide a comprehensive scientific analysis of published studies within 2000 to 2022 years. The Web of Science Core Collection (WoSCC) database, one of the most comprehensive sources of literature, provides users with online access to high-quality literature and is an important component for the bibliometric analysis of published literature. With the help of 2 bibliometric visualization tools, CiteSpace 6.1.R2 (created by Prof Chaomei Chen in 2004) and VOSviewer 1.6.17.0, we mapped the knowledge graph of acupuncture for cancer pain from 2000 to September 2022. Data visualization was performed based on the bibliometric analysis of annual publication trends, countries, authors, research institutions, keywords, and co-citation network citations.

## 2. Materials and Methods

### 2.1. Search strategy

We used the WoSCC to organize documents related to the use of acupuncture for the management of CRP. The search strategy included acupuncture or its synonyms (subject), cancer or its synonyms and pain or pain relate (subject). The publication period was restricted to 2000 to 2022 (retrieved on September 4, 2022). We finally included 747 articles as original literature. The specific search strategy is shown in Table 1.

**Table 1:** Search strategy.

ID	Result	Search strategy
#1	21,744	TS= (“Acupuncture” OR “Acupuncture Therapy” OR “Acupuncture, Ear” OR “Acupuncture Points” OR “Acupuncture Analgesia”)
#2	3,963,559	TS= (“Tumor” OR “Neoplasm” OR “Tumors” OR “Neoplasia” OR “Neoplasias” OR “Cancer” OR “Cancers” OR “carcinoma” OR “neoplasm”)
#3	684,669	TS= (“Pain” OR “Painful” OR “Ache” OR “Pains” OR “Related pain”)
#4	747	^#^1 and ^#^2 and^#^3

### 2.2. Inclusion criteria

**Figure 1.**
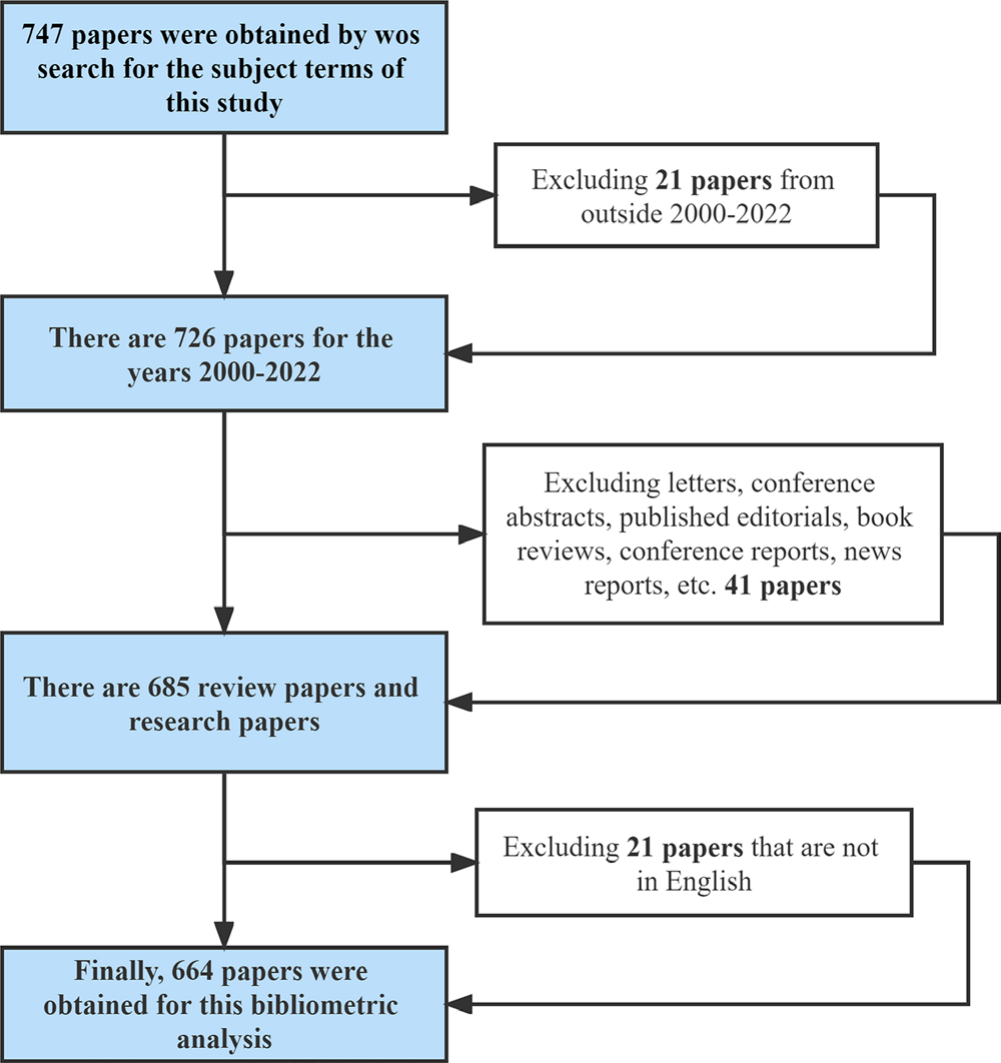
Flowchart of data inclusion and exclusion criteria.

Includes pain and pain-related, acupuncture and cancer articles and reviews from different academic journals. We excluded letters, conference abstracts, published editorials, book reviews, conference presentations, news reports, etc. We included literature in English and did not specify restrictions on species. Finally, we included 664 articles. Inclusion criteria are shown in the flowchart Figure 1.

### 2.3. Bibliometric analysis

According to the system internal function of WoSCC, following the download of the details relating to the distribution of countries/regions, year of publication, institutions, journals, authors, and keyword related files, we used office16 to sort and analyze publications. CiteSpace was used for identifying the collaborative author network of countries/institutions, double map analysis, and keyword detection with citation explosion. Citation explosion indicated an increase of hot topics for potential work in the field over a certain period of time, which was an important indicator for determining research trends.

### 2.4. Bibliometric analysis terminology

Knowledge graph visualization is a product of the development of graphic data technology. Data visualization is the aggregation of available information into a large network that contains a semantic model of the data for users to query and explore. In this way, the raw data are converted into graphical information and the information is presented in a more visual and intuitive way. Intermediate centrality is the number of times a node is located on the shortest route between other nodes, and it is one of the key indicators of the importance of a node. It is generally considered that a node with intermediate centrality greater than 0.1 is a critical node. The more times a node is located on the shortest path, the higher the centrality of the node.

### 2.5. Bibliometric analysis software

We used 2 software programs for the bibliometric analysis and visual analysis of the related data: CiteSpace 6.1.R2 and VOSviewer 1.6.17.0. VOSviewer was applied to the network consisting of authors, keywords, countries, research organizations and their density maps by using counting methods. CiteSpace 6.1.R2 was used to describe the collaborative networks in this research area.

## 3. Results

### 3.1. Publication trend

**Figure 2.**
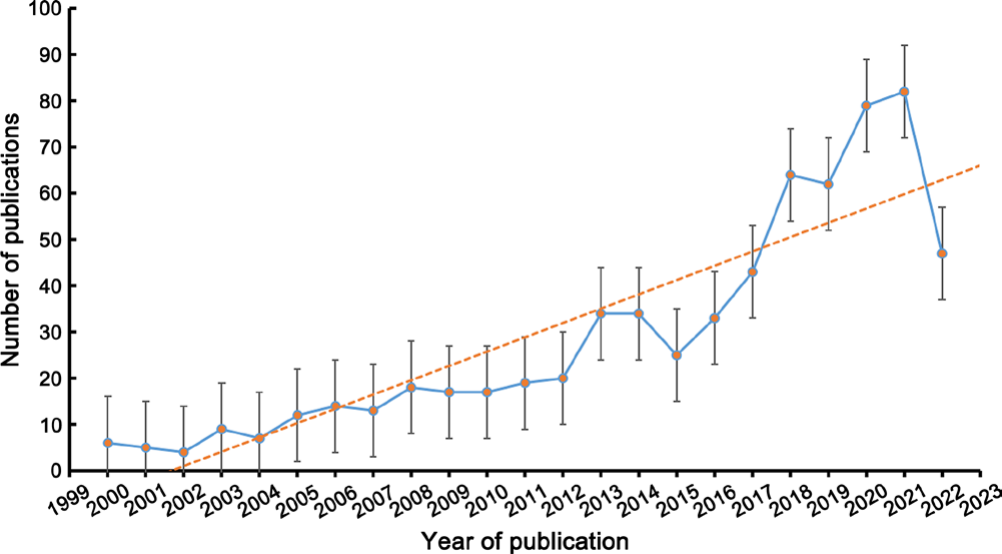
A line chart of the number of publications published each year, 2000 to 2022.

Figure 2 illustrates a general upward trend in overall publications over the period 2000 to 2022. There was no growth in the number of articles published in the period 2000 to 2002. The number of articles published grew slowly during 2003 to 2012 period and grew rapidly during 2013 to 2021 period. There were some fluctuations between years, for example in 2015 and 2019. From the 6 articles in 2000 to the 82 articles in 2021, with an additional 47 articles were published in 2022 as of September 4, 2022. Over the past 22 years, the number of publications on acupuncture for CRP tended to grow at a stagnant rate in the early phase (2000–2005), at a slow rate in the middle phase (2006–2012), and at a rapid rate in the later phase (2013–2021). Our linear regression model prediction (*Y* = 3.0958X-6196.9, *R*^2^ = 0.7951) analysis suggests that the field is currently in a phase of steady growth for global publication production.

### 3.2. Analysis of countries or regions

**Figure 3.**
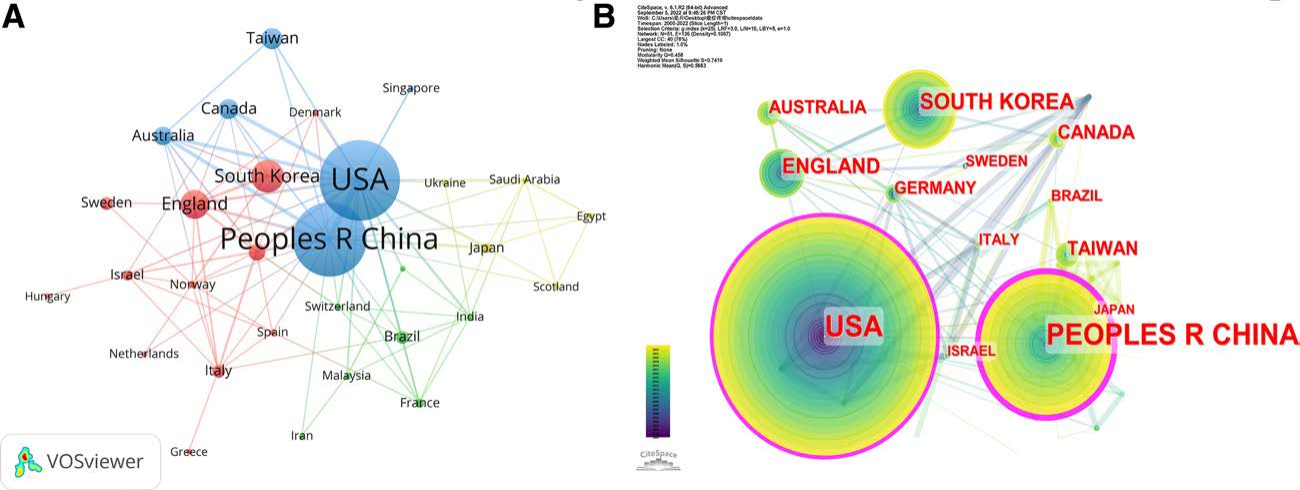
(A) The co-occurrence map of countries or regions. The color of the nodes represents clustering and the size of the nodes represents the number of articles and lines between nodes represent the strength of collaborations. (B) The centrality of countries or regions. The area of the circle on the outer edge of the nodes represents the centrality of the countries or regions.

The top 10 productive countries are shown in Table 2 and in Figure 3 we analyzed by setting a sieve condition (countries that published at least 2 papers). There were 30 items, that is, a map of cooperative network relationships constructed by 30 countries or regions with each other (Fig. 3A), and the top 3 ranked were the United States, the People’s Republic of China, and South Korea. Among them, the United States published a large number of articles about acupuncture for cancer pain, with 244 articles, 20 links, and a total linkage intensity of 97. This was followed by the People’s Republic of China, which published 215 articles, 19 links with other countries, and a total linkage intensity of 72. The next most important country was South Korea, which published 59 papers, 5 links with other countries, and a total linkage intensity of 17. This indicates that the U.S. publishes more articles than the People’s Republic of China and South Korea, and the intensity of cooperation between the U.S. and other countries is the strongest. As depicted in Figure 3B, the larger the area of the edge circle, the greater the centrality the country is. The centrality of the People’s Republic of China (0.48) is greater than the centrality of the U.S. (0.39). However, the U.S. has a weaker influence than the People’s Republic of China despite the number of articles published. This means that the People’s Republic of China cooperates more closely with other countries.

**Table 2:** Top 10 most productive countries or regions for publication number.

Rank	Country or regions	Publication number	Centrality
1	USA	244	0.39
2	Peoples R China	215	0.48
3	South Korea	59	0.00
4	England	49	0.05
5	Taiwan (Peoples R China)	30	0.00
6	Canada	28	0.01
7	Australia	25	0.01
8	Germany	21	0.05
9	Brazil	14	0.08
10	Italy	14	0.09

### 3.3. Analysis of institutions

**Figure 4.**
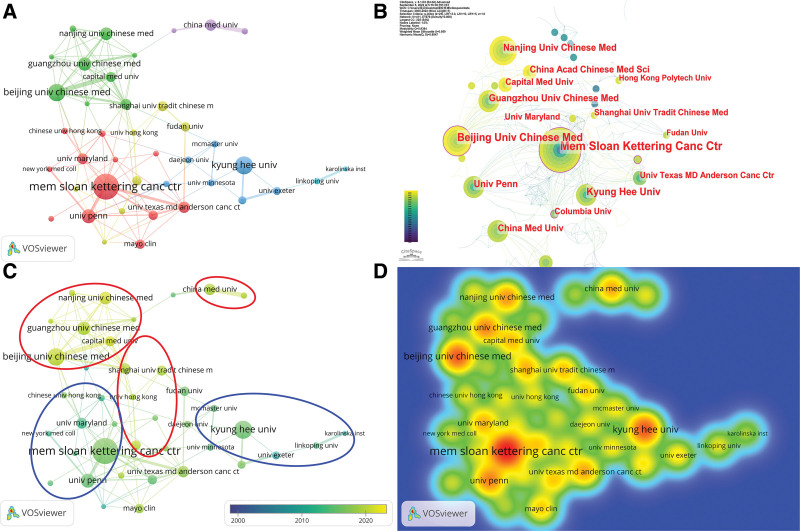
(A) The co-occurrence map of institutions. The color of the nodes represents clustering and the size of the nodes represents the number of articles and lines between nodes represent the strength of collaborations. (B) The centrality of institutions. The area of the circle on the outer edge of the nodes represents the centrality of the countries or regions. (C) The timeline visualization of institutions. (D) The co-occurrence density map of institutions. The higher the intensity of the red color node, the higher the number of articles.

Out of the 944 institutions affiliated with the publications, we filtered them by setting the condition of at least 6 published articles. Finally, 41 institutions were obtained for the subsequent analysis. Using the number of publications as the basis for analysis and combining the Figure 4D density plot and Table 3, we can see that Memorial Sloan-Kettering Cancer Center published the first number of articles, and the second and third were Kyung Hee University and Beijing University of Chinese Medicine, respectively. Among the top 10 publishers, the top 5 cited institutions were Memorial Sloan-Kettering Cancer Center (1485 Citations), China Medical University (818 Citations), University of Maryland (709 Citations), University of Pennsylvania (651 Citations) and Kyung Hee University (510 Citations). We found there is not necessarily a link between the number of publications and the number of citations.

**Table 3:** Top 10 most productive institutions.

Rank	Document	Institution	Centrality	Citations	Total link strength
1	37	Mem Sloan Kettering Canc Ctr	0.16	1458	25
2	25	Kyung Hee Univ	0.05	510	6
3	24	Beijing Univ Chinese Med	0.11	198	22
4	19	Univ Penn	0.04	651	16
5	18	Guangzhou Univ Chinese Med	0.04	145	14
6	17	Nanjing Univ Chinese Med	0.01	75	11
7	15	China Med Univ	0.02	818	11
8	15	Univ Texas Md Anderson Canc Ctr	0.02	125	16
9	14	China Acad Chinese Med Sci	0.05	180	18
10	14	Univ Maryland	0.17	709	8

Combining centrality and total link strength for analysis (Fig. 4A; Table 3), we found that the top 3 institutions in terms of centrality were University of Maryland (0.17), Memorial Sloan-Kettering Cancer Center (0.16) and Beijing University of Chinese Medicine (0.11), and their total link strength were 8, 25 and 22 respectively. The results showed that there was not necessarily a connection between centrality and link strength.

In Figure 4B and Table 3, the centrality of University of Maryland, Memorial Sloan-Kettering Cancer Center and Beijing University of Chinese Medicine were 0.17, 0.16, 0.11 respectively. In the top 10 institutions, we found that University of Maryland has a higher impact. However, combining the number of articles, centrality, number of citations and link strength, it is undoubtedly Memorial Sloan-Kettering Cancer Center has the strongest influence.

Figure 4C shows the trend of the number of publications in the timeline, and we find that the institutions in the red circle are at the forefront of research, and most of them are Chinese domestic traditional Chinese medicine (TCM) universities. The institutions in the blue circles are in a slow state of research, and most of them are foreign cancer research centers and foreign comprehensive universities. This may be due to the love of research on acupuncture as a traditional Chinese medicine in Chinese domestic TCM universities.

### 3.4. Analysis of authors

#### 3.4.1. Analysis of author collaboration.

The top 10 authors in terms of number of publications and their citations, Mao Jun J. was the most successful author (Supplemental Digital Content 1, http://links.lww.com/MD/J483), while Heather Greenlee had only 7 papers but obtained 867 citations, indicating the high quality of Greenlee’s research in the field. This suggests that there is not necessarily a link between the number of published papers and their quality.

**Figure 5.**
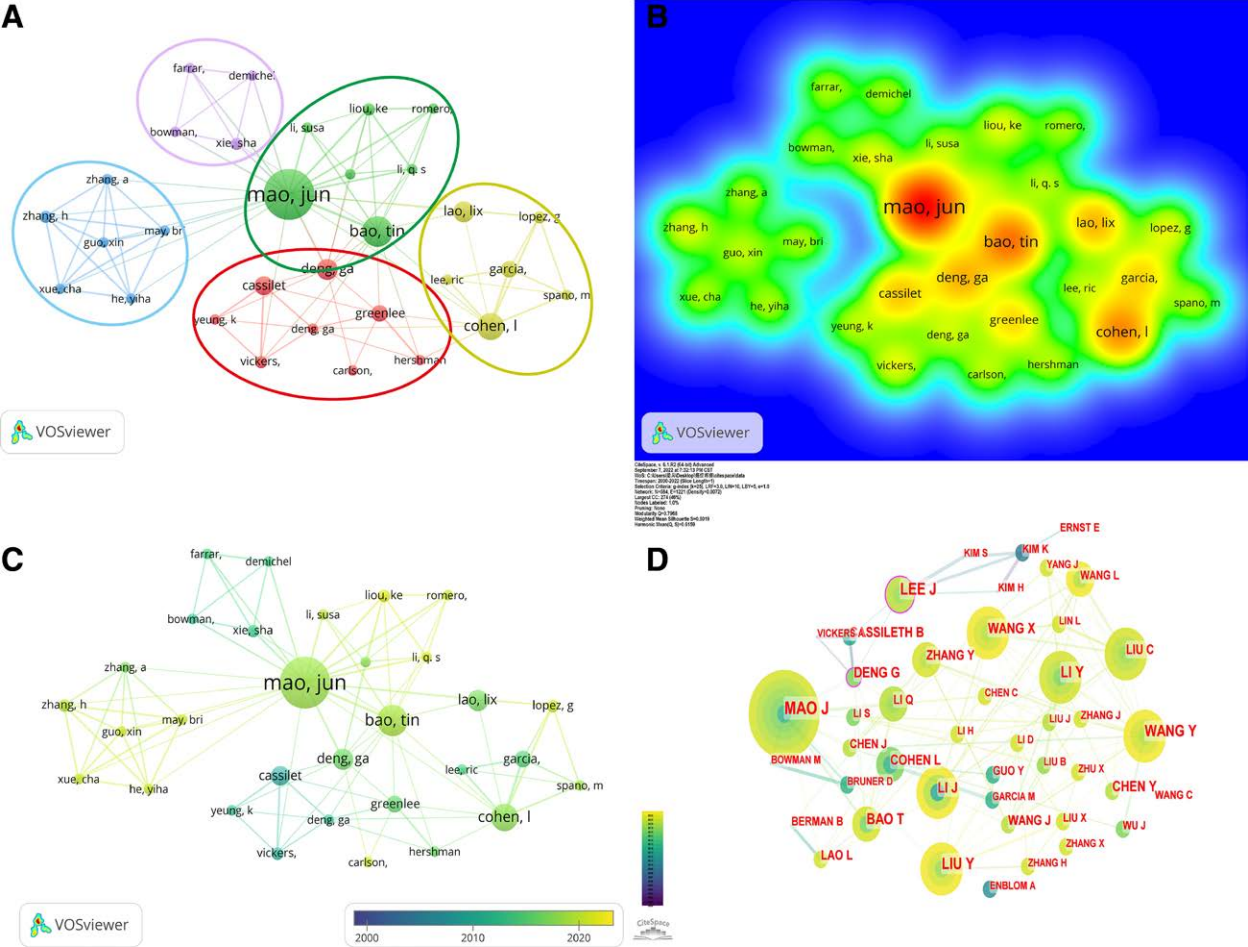
(A) The co-occurrence map of authors. (B) The co-occurrence density map of authors. (C) The timeline visualization of authors. (D) The centrality of authors.

In Figure 5A, we have clustered the author network relationship map. There are 5 clusters, blue, yellow, purple, red and green, representing 5 different research teams. A larger link point in a cluster represents a greater number of publications and influence in that cluster. In density map, the warmer the node color, the more articles are published by the author. As depicted in Figure 5B, Mao Jun J., Bao Ting and Cohen Lorenzo published the top 3 number of articles. Figure 5C shows the timeline of the authors research, with the linking node closer to yellow indicating that the author is at the forefront of the timeline, and the linking node closer to blue indicating that the author is behind in the timeline. Figure 5D shows that both author Lee J and author Deng G have a centrality of 0.11, indicating that Lee J and Deng G are the most influential of all authors in the field of acupuncture for cancer pain (Supplemental Digital Content 2, http://links.lww.com/MD/J484).

#### 3.4.2. Analysis of co-cited authors.

**Figure 6.**
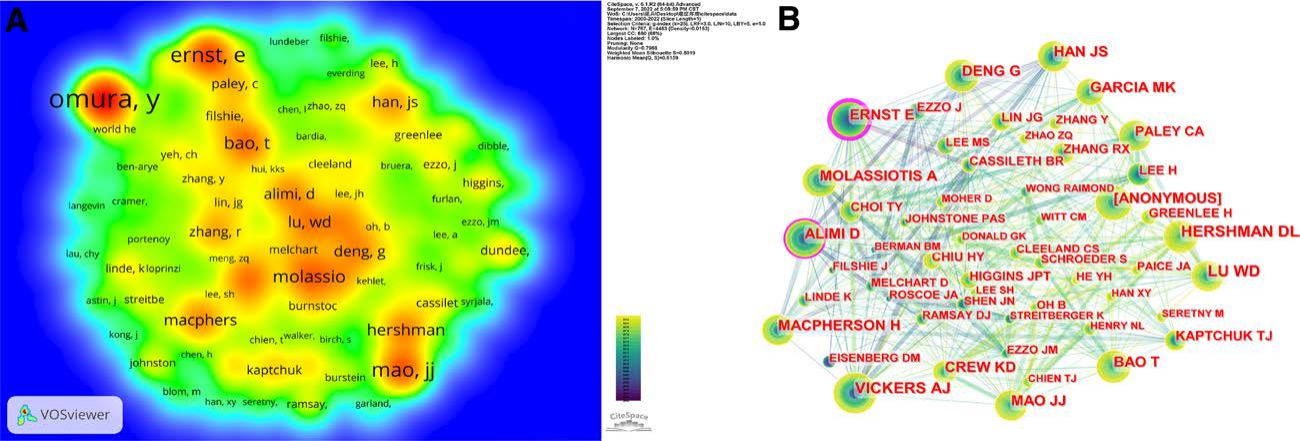
(A) The co-occurrence density map of co-cited author. The higher the intensity of the red color node, the higher the number of citations. (B) The co-occurrence map and centrality of co-cited author.

Table 4 shows the number of co-citations and centrality of the top 10 authors. Combining Table 4 with the density plot Figure 6A (the darker the red, the higher number of citations), we find that Omura Y has the highest number of citations at 248 and a centrality of 0.00. However, Ernst E, Alimi D and Molassiotis A have a centrality of 0.22, 0.14 and 0.10 respectively. And all the 3 authors have fewer citations than Omura Y. Figure 6B suggests that the articles published by Ernst E, Alimi D and Molassiotis A have a certain influence and but are not directly affected by the number of citations.

**Table 4:** Top 10 co-cited authors.

Rank	Cited author	Citations	Centrality
1	Omura, Y	248	0.00
2	Ernst, E	178	0.22
3	Mao, JJ	163	0.05
4	Molassiotis, A	128	0.10
5	Bao, T	127	0.08
6	Vickers, AJ	120	0.07
7	Hershman, DL	115	0.04
8	Lu, WD	107	0.03
9	Macpherson, H	106	0.04
10	Alimi, D	100	0.14

### 3.5. Analysis of journals

**Figure 7.**
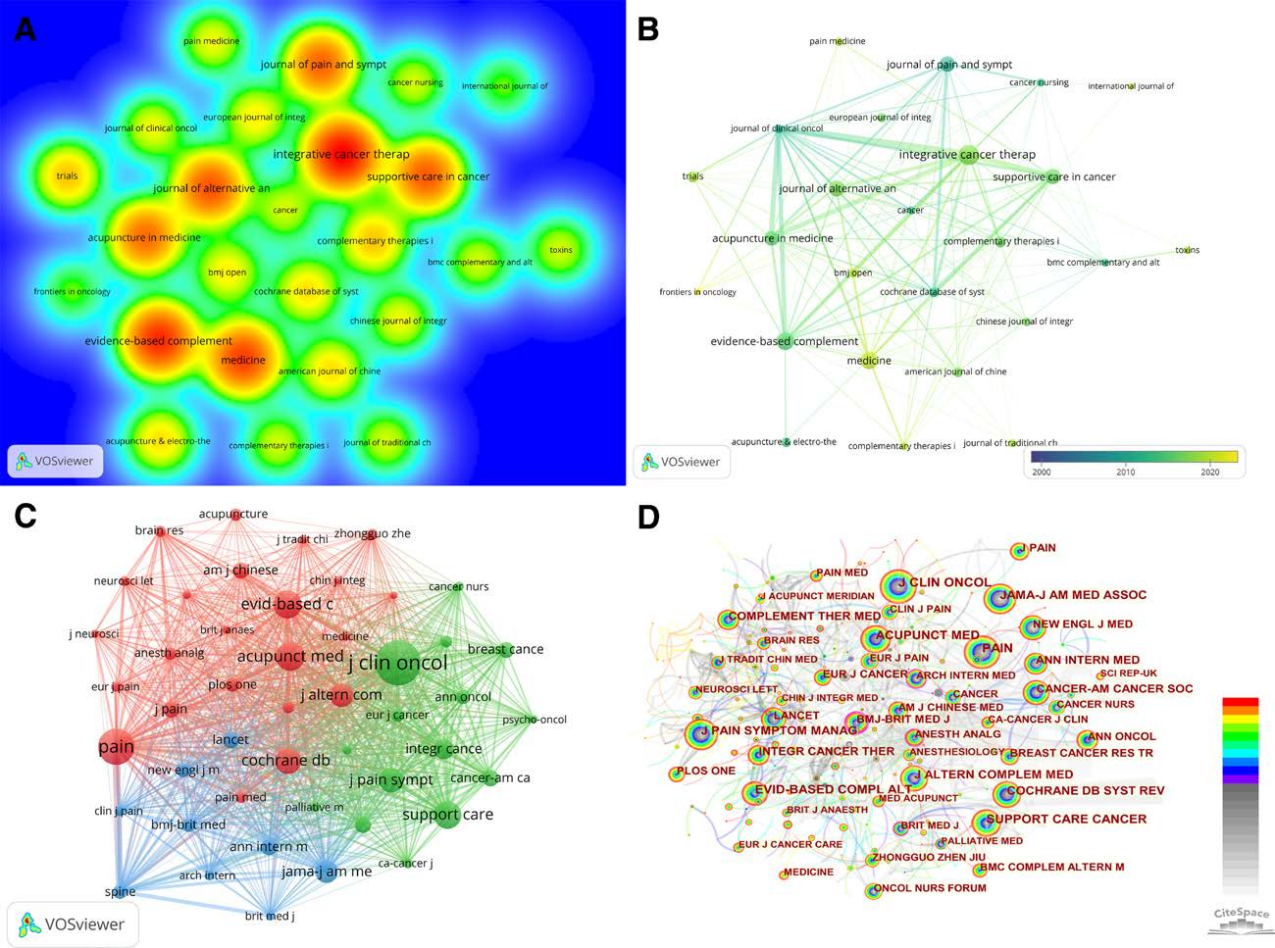
(A) The co-occurrence density map of journals. The higher the intensity of the red color node, the higher the number of articles. (B) The timeline visualization of journals. (C) The co-occurrence map of co-cited journals by VOSviewer. (D) The co-occurrence map and centrality of co-cited journals by CiteSpace.

A total of 250 academic journals published articles on acupuncture for cancer pain. Figure 7 illustrated the large number of articles had published in journals related to cancer, evidence-based medicine, complementary and alternative medicine, pain management and acupuncture. As shown in Figure 7A, the warmer the color of the nodes in the density plot is, the higher number of co-citations are in the journals. This suggests that the topic of acupuncture for cancer pain is more focused on clinical research, evidence-based medical research and integrative medical research. Journal of Pain and Symptom Management has the strongest color, indicating the highest level of impact and is ranked first. Table 5 lists the top 10 journals that have published the most papers on acupuncture for cancer pain and shows the number of articles, number of citations, average number of citations, and impact factor for the top 10 journals. As shown in Table 5, the top 3 journals in terms of number of published articles are Integrative Cancer Therapies, Evidence Based Complementary and Alternative Medicine and Medicine. We found an interesting phenomenon that the Journal of Pain and Symptom Management (impact factor [IF]: 5.576), with an average number of citations of 44.55, published 22 articles and had the highest number of citations of 980, while the two journals with the lowest average number of citations were Medicine (IF: 1.817) and Trials (IF: 2.728).

**Table 5:** Top 10 most productive journals.

Rank	Journal	Documents	Citations	Average citations	Impact factor
1	Integrative Cancer Therapies	36	458	12.72	3.077
2	Evidence-based Complementary and Alternative Medicine	30	498	16.60	2.65
3	Medicine	27	118	4.37	1.817
4	Journal of Alternative and Complementary Medicine	24	399	16.63	2.381
5	Supportive Care in Cancer	23	513	22.30	3.359
6	Journal of Pain and Symptom Management	22	980	44.55	5.576
7	Acupuncture in Medicine	21	282	13.43	1.976
8	Complementary Therapies in Medicine	12	148	12.33	3.335
9	Trials	12	49	4.08	2.728
10	Acupuncture & Elcetro-Therapeutics Research	10	108	10.80	0.684

Figure 7B illustrated the trend of journals in the timeline. We find that Frontiers in Oncology is at the forefront of research in the timeline, followed by Pain Medicine, Medicine, BMJ Open and Complementary Therapies in Clinical Practice. Figure 7C and D both are co-cited journals graphs, and the top 10 journals in terms of number of citations and centrality of co-cited journals by different software are shown in Table 6. The top journal in terms of number of articles published is Integrative Cancer Therapies (36 documents), the top journal in terms of number of citations is J Clin Oncol (1106 citations), and the journal with the highest citation centrality is BMJ-Brit Med J (0.3).

**Table 6:** Top 10 most cited journals with the highest citations and centrality.

Rank	Citations	Total link strength	Cited journal	Centrality	Cited journal
1	1106	28,442	J Clin Oncol	0.3	Bmj-Brit Med J
2	814	26,055	Pain	0.2	Neuroimage
3	605	14,457	Acupunct Med	0.19	Arch Intern Med
4	579	17,729	Evid-Based Compl Alt	0.18	Brit Med J
5	559	16,096	Support Care Cancer	0.15	Anesth Analg
6	512	15,651	Cochrane Db Syst Rev	0.14	Rheumatology
7	477	12,360	J Pain Symptom Manag	0.14	Am J Med
8	444	13,032	J Altern Complem Med	0.13	Acupuncture Electro
9	435	13,079	Jama-J Am Med Assoc	0.13	Am J Obstet Gynecol
10	371	11,767	Integr Cancer Ther	0.12	Arch Women Ment Hl Th

### 3.6. Analysis of references

**Figure 8.**
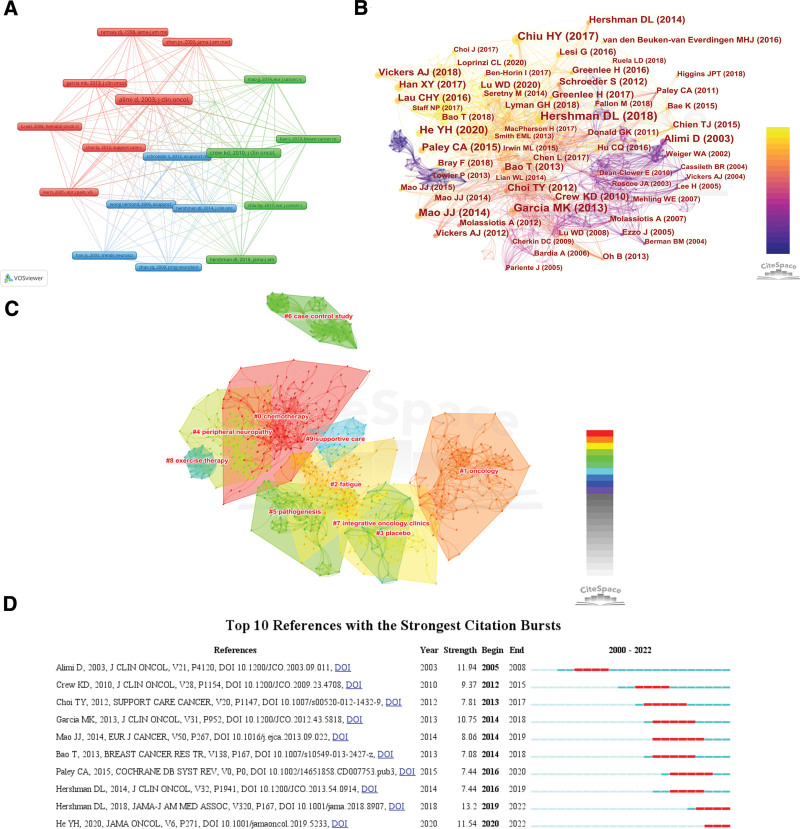
(A) The co-occurrence map of co-cited references by VOSviewer. (B) The co-occurrence map of co-cited references by CiteSpace. (C) Cluster map of co-cited references. (D) Map of the top 10 references with the strongest citation bursts.

Reference co-citations are two (or more) articles that are cited in one or more articles at the same time, which can be used to assess the degree of relevance between articles. In addition, reference co-citations highlight key articles that have contributed to research in the field. According to the network of co-cited references in VOSviewer, the relevance of the references can be presented by the distance between two references or the color of the node. As shown in Figure 8A, the cited references can be divided into 3 categories. Figure 8B shows the reference co-citation map consisting of 889 cited references and 3399 co-citation links from 2000 to 2022. Tables 7 and 8 lists the top 10 most cited references and the highest centrality references, respectively.

**Table 7:** Top 10 most cited references with the highest frequency.

Rank	Frequency	Cited reference	Representative author (publication year)	DOI
1	36	JAMA-J AM MED ASSOC, V320, P167	Hershman DL (2018)	10.1001/jama.2018.8907
2	30	EUR J CANCER CARE, V26, P0	Chiu HY (2017)	10.1111/ecc.12457
3	29	J CLIN ONCOL, V31, P952	Garcia MK (2013)	10.1200/JCO.2012.43.5818
4	27	JAMA ONCOL, V6, P271	He YH (2020)	10.1001/jamaoncol.2019.5233
5	23	EUR J CANCER, V50, P267	Mao JJ (2014)	10.1016/j.ejca.2013.09.022
6	22	COCHRANE DB SYST REV, V0, P0	Paley CA (2015)	10.1002/14651858.CD007753.pub3
7	21	J CLIN ONCOL, V21, P4120	Alimi D (2003)	10.1200/JCO.2003.09.011
8	19	J PAIN, V19, P455	Vickers AJ (2018)	10.1016/j.jpain.2017.11.005
9	18	J CLIN ONCOL, V28, P1154	Crew KD (2010)	10.1200/JCO.2009.23.4708
10	18	SUPPORT CARE CANCER, V20, P1147	hoi TY (2012)	10.1007/s00520-012-1432-9

**Table 8:** Top 10 most cited references with the highest centrality.

Rank	Centrality	Cited reference	Representative author (publication year)	DOI
1	0.46	J PAIN SYMPTOM MANAG, V33, P258	Mehling WE (2007)	10.1016/j.jpainsymman.2006.09.016
2	0.25	AM J HOSP PALLIAT ME, V27, P446	Alshemmari S (2010)	10.1177/1049909110362438
3	0.23	J PAIN SYMPTOM MANAG, V28, P244	Cassileth BR (2004)	10.1016/j.jpainsymman.2003.12.016
4	0.20	Br Homeopath J, V89, P8	Balzarini A (2000)	10.1054/homp.1999.0328
5	0.19	J CLIN ONCOL, V28, P1154	Crew KD (2010)	10.1200/JCO.2009.23.4708
6	0.18	J CLIN ONCOL, V32, P1840	Bower JE (2014)	10.1200/JCO.2013.53.4495
7	0.18	CHIN J INTEGR MED, V20, P136	Lian WL (2014)	10.1007/s11655-013-1439-1
8	0.17	J Soc Integr Oncol, V7, P4	Balk Judith (2009)	None
9	0.17	ACUPUNCT MED, V31, P264	Oh B (2013)	10.1136/acupmed-2012-010309
10	0.16	3 INT C NEUR PAIN, V4, P128	AKHILESWARAN R (2010)	None

Cluster analysis of the co-cited references was performed to analyze common themes in similar articles. Figure 8C shows the 10 clusters, and the first 5 clusters are #0 chemotherapy, #1 oncology, #2 fatigue, #3 placebo and #4 peripheral neuropathy. Figure 8D shows the top 10 references with the strongest citation bursts. The article published by Alimi D, J CLIN ONCOL, V21, P4120 (2003) has the highest strength value (11.94), followed by He YH JAMA ONCOL, V6, P271 (2020) (11.54) and Garcia MK J CLIN ONCOL, V31, P952 (2013) (10.75).

### 3.7. Analysis of keywords

#### 3.7.1. Analysis of keywords co-occurrence.

**Figure 9.**
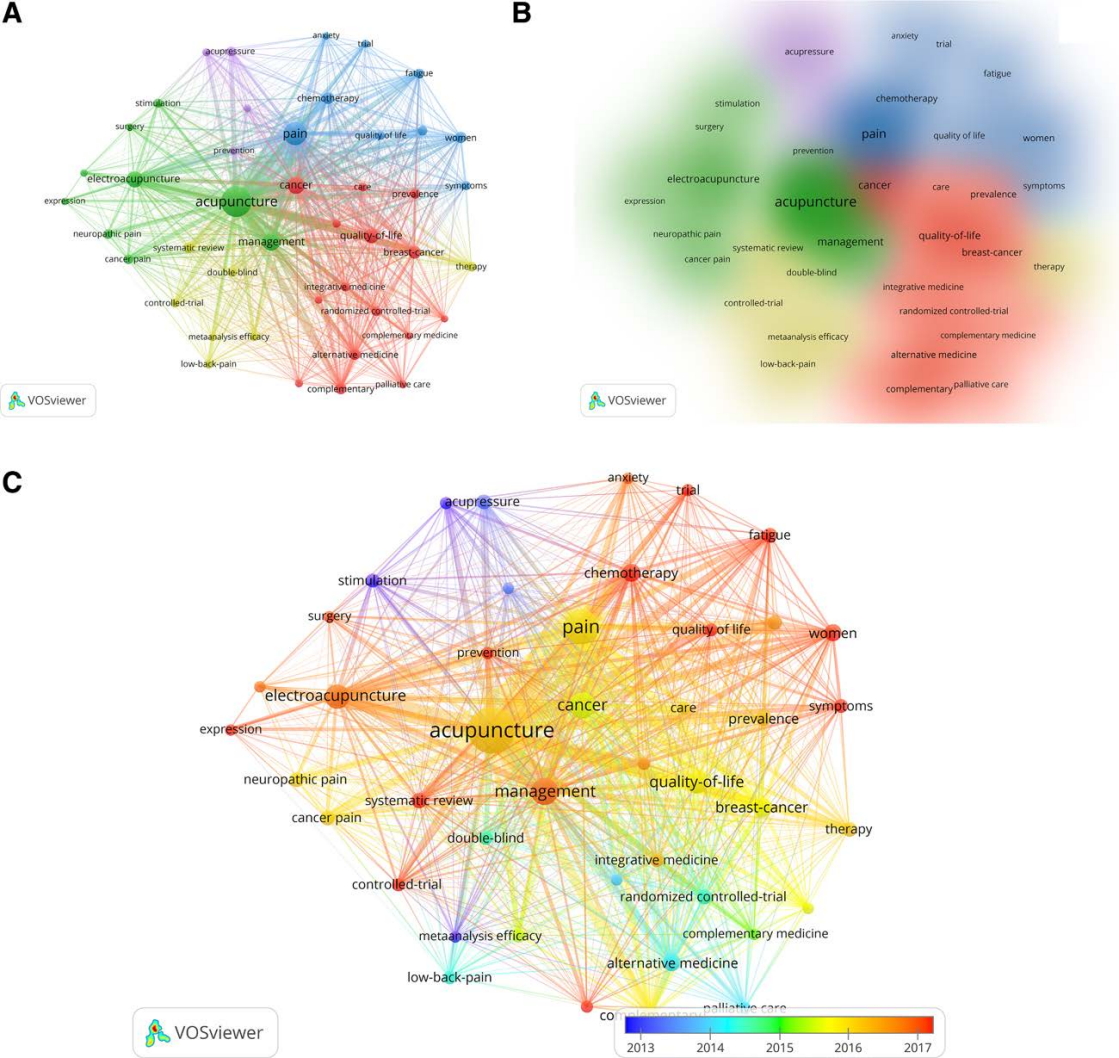
(A) The co-occurrence map of keywords by VOSviewer. (B) The co-occurrence density map of keywords. (C) The timeline visualization of keywords.

**Figure 10.**
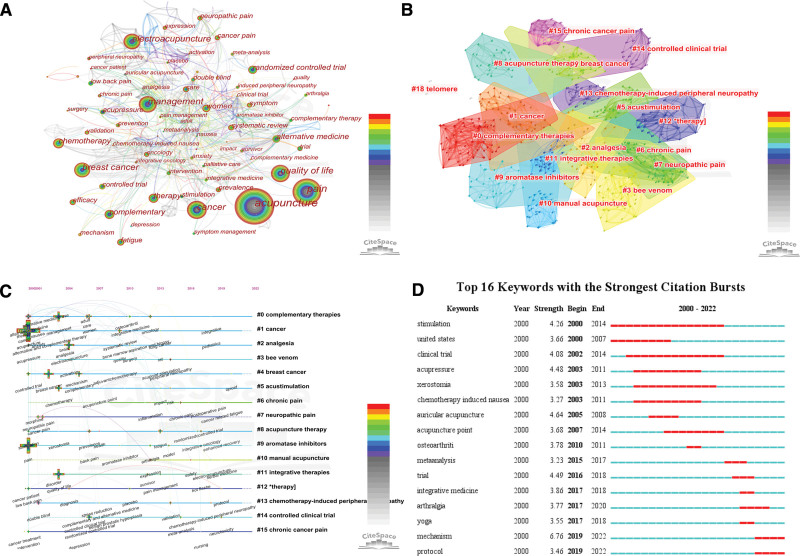
(A) The co-occurrence map of keywords by CiteSpace. (B) Cluster map of keywords. (C) Timeline view of keywords. (D) Map of the top 14 keywords with the strongest citation bursts.

Keywords co-occurrence analysis can identify the research hotspots and trends. VOSviewer identified a total of 2564 keywords. By setting the minimum number of occurrences of keywords to 25, 44 keywords in total have been analyzed. As shown in Figure 9A and B, the keywords could be divided into 5 clusters, the green cluster was represented by the terms “acupuncture, electroacupuncture and management,” the blue cluster was represented by “pain, fatigue, women,” the red cluster was represented by “cancer, breast cancer, quality of life,” the yellow cluster was represented by “controlled-trail, systematic review” and the purple cluster was represented by “acupressure and prevention.” We further analyze these keywords. Figure 9C illustrated the research hot topics of keywords during 2000 to 2022. We have set the period 2013 to 2017 in order to better observe the research trends in the timeline. Table 9 shows the top 10 keywords with the highest citations and centrality. Acupuncture has the highest citation of 407 and alternative medicine has the highest centrality (0.29). From Figure 10A, the keyword co-occurrence graph by CiteSpace, we can know that the main keywords in the studies related to acupuncture for cancer pain are acupuncture (398 times), pain (224 times), management (129 times), cancer (129 times), quality of life (116 times), electroacupuncture (113 times), breast cancer (111 times), chemotherapy (62 times), therapy (60 times), complementary (56 times), randomized controlled trial (54 times) and women (52 times). And these keywords are the latest topics. In general, the cancer patients, mainly women with breast cancer, need acupuncture to manage the complications associated with cancer or cancer chemotherapy, including pain, fatigue, depression and anxiety.

**Table 9:** Top 10 keywords with the highest citations and centrality.

Rank	Citation counts	Keywords	Centrality	Keywords
1	407	acupuncture	0.29	alternative medicine
2	230	pain	0.24	double blind
3	135	management	0.18	breast cancer
4	132	cancer	0.13	cancer pain
5	118	electroacupuncture	0.13	complementary therapy
6	95	quality-of-life	0.13	analgesia
7	74	breast-cancer	0.13	acupuncture point
8	64	chemotherapy	0.12	clinical trial
9	59	alternative medicine	0.11	activation
10	59	complementary	0.11	outcm

Overall, it demonstrates the prominent role of acupuncture in TCM as an integrated medicine and a complementary alternative medicine. Traditional acupuncture and electroacupuncture play an important role as alternative therapies to relieve pain and improve the quality of life of cancer patients. The main types of cancer pain treated with acupuncture are breast pain, neuralgia and low back pain, which are commonly characterized by chronic pain.

#### 3.7.2. Analysis of keywords clustering map.

Log-likelihood ratio was calculated for the keywords and 15 keyword clustering labels were obtained based on the clustering map of keywords co-occurrence. The development pattern and future research direction of acupuncture for cancer pain can be explored from the cluster map. Modularity reflects the degree of connection between the nodes within the clusters with a value of 0.7412, and Silhouette reflects the homogeneity of the network with a value of 0.8845, which indicates that the clustering is effective and the clustering consistency is high with good homogeneity results.

As shown in Figure 10B, the 15 clusters are #0 Complementary therapies, #1 Cancer, #2 analgesia, #3 bee venom, #4 breast cancer, #5 acustimulation, #6 chronic pain, #7 neuropathic pain, #8 acupuncture, #9 aromatase inhibitors, #10 manual acupuncture, #11 integrative therapies, #12 therapy, #13 chemotherapy- induced peripheral neuropathy, #14 controlled clinical trial and #15 chronic cancer pain, which are the most discussed topics in the field. In Table 10 are the popular academic terms labeled for each cluster. The smaller the cluster ordinal number, the more nodes it contains, indicating that the more keywords it contains, the higher the heat of the cluster research; the larger Silhouette value indicates that the cluster is more critical in the field. From the clustering labels, it can be seen that acupuncture, as a complementary alternative therapy, acts on cancer pain mostly for the purpose of evaluating the clinical effectiveness of acupuncture therapy for analgesia, and the focus of the study is mostly on the clinical symptoms related to cancer patients that were significantly improved by controlled clinical trials of acupuncture, as well as the differences in the clinical efficacy of the therapy.

**Table 10:** The popular academic terms labeled for each cluster.

Cluster ID	Size	Silhouette	Mean (yr)	Top terms (LLR)
#0	56	0.882	2009	Complementary therapies; adult; alternative medicine use; integrative medicine; women
#1	46	0.865	2007	Cancer; acupuncture; children; palliative care; systematic review
#2	43	0.788	2010	Analgesia; electroacupuncture; auricular acupuncture; cytokines; transcutaneous electrical acupoint stimulation
#3	43	0.935	2013	Bee venom; paclitaxel; mechanical allodynia; melittin; ATP
#4	35	0.898	2007	Breast cancer; chemotherapy induced nausea; prevention; antiemetics; p6
#5	35	0.978	2009	Acustimulation; telomere; DHEA; TXB2; integrin alpha
#6	31	0.851	2016	Chronic pain; inflammation; adverse events; yoga; vincristine
#7	31	0.931	2007	Neuropathic pain; chronic constriction injury; cancer pain; hyperalgesia; neural regeneration
#8	30	0.797	2012	Acupuncture therapy; cancer survivors; integrative oncology; ESAS; Tibetan herbal patch
#9	30	0.868	2013	Aromatase inhibitors; arthralgia; real; tenosynovial change; acupressure
#10	29	0.817	2019	Manual acupuncture; electro-acupuncture; electroacupuncture; nociception; microglia
#11	29	0.886	2015	Integrative therapies; mindfulness; quality of life; breast cancer survivor; insomnia
#12	27	0.917	2008	*Therapy; protocol; opioid-induced constipation; humans; placebo
#13	24	0.924	2009	Chemotherapy-induced peripheral neuropathy; cancer related pain; complementary and alternative medicine; extended release; ankylosing spondylitis
#14	20	0.907	2015	Controlled clinical trial; randomized controlled trial; meta-analysis; opioid-induced constipation; oncology nursing
#15	19	0.943	2006	Chronic cancer pain; patient education; cancer treatment; aromatherapy massage; triglyceride

LLR = log-likelihood ratio.

#### 3.7.3. Analysis of keywords timeline view and burst.

A timeline view of keywords was generated using the cluster name as the vertical axis and the year of publication as the horizontal axis. As shown in Figure 10C, in the time frame discussed in this paper, many scholars had already conducted research on the treatment of cancer pain with acupuncture in 2000, and it can be inferred that this academic field emerged in the 1990s or even earlier, and more and more researchers and scholars have joined in this field, which indicates that the heat of acupuncture treatment for cancer pain has always existed in the world.

The keywords emergent words refer to the keywords that appear within a short period of time. And this information can show the research hotspots over time, indicate research trends in recent years and possibly foreshadow the future research trends. Figure 10D shows the top 16 keywords with the strongest citation bursts from 2000 to 2022. They are stimulation, united states, clinical trial acupressure, xerostomia, chemotherapy induced nausea, auricular acupuncture, acupuncture point, osteoarthriti, meta-analysis, trial, integrative medicine, arthralgia, yoga, mechanism and protocol. Among these keywords, we found that the strength of stimulation (4.26) and clinical trials (4.08) are not the highest among the 16 keywords, but “stimulation” lasted for 15 years (2000–2014) and “clinical trials” lasted for 13 years (2002–2014). This indicates that acupuncture stimulation in clinical trials has continued to be a hot topic for most of the period from 2000 to 2022. In addition, the burst keywords in the last 3 years were “mechanism” and “protocol,” and mechanism ranking the highest among the 16 keywords in terms of strength (6.76). This indicates that the mechanism of acupuncture for cancer pain and the experimental protocol of acupuncture clinics have attracted more attention from researchers in recent years.

## 4. Discussion

In this age of big data, researchers need to understand the development trends of CRP related research. The bibliometric analysis can use several software tools to analyze and visualize existing literature comprehensively, also to understand the research trends and predict the frontiers of CRP research.

### 4.1. General information

Since 2000, concurrent with the burgeoning of socioeconomic development, the number of cancer patients has increased year by year. Furthermore, cancer patients may experience pain at different stages, and the side effects of opioids and nerve block modalities for pain treatment not only fail to improve the effectiveness of medical treatment, but also affect the quality of life and survival of cancer patients. In this paper, the years 2000 to 2022 are used as an example. The number of articles published during the 2000 to 2012 period grew slowly, and from 2013 to 2021 it grew rapidly. There are fluctuations in some specific years, but the overall number of publications relating to acupuncture for cancer pain has steadily increased. The top 3 countries in terms of the number of published articles are the United States (244 articles), the People’s Republic of China (215 articles), and South Korea (59 articles). Among them, the People’s Republic of China (centrality is 0.48) has the highest impact.

For institutions, the top three with the highest number of publications relating to acupuncture for cancer pain are the Memorial Sloan-Kettering Cancer Center, the Kyung Hee University, and the Beijing University of Chinese Medicine. However, the University of Maryland with 14 publications is the most influential (centrality = 0.17), followed by the Memorial Sloan-Kettering Cancer Center (centrality = 0.16) and the Beijing University of Chinese Medicine (centrality = 0.11). From the overall timeline, most Chinese medical universities in the People’s Republic of China are at the forefront of research, while foreign cancer research centers and foreign comprehensive universities are relatively backward.

For authors, Mao Jun J. has the highest number of published articles, while Heather Greenlee’s 7 articles obtained 867 citations, indicating the high quality of Greenlee’s research in the field. This suggests that there is not necessarily a link between the number of published papers and their quality. The centrality of both author LEE J and author DENG G was 0.11, indicating that LEE J and DENG G are the most influential of all authors in the field of acupuncture for cancer pain. In terms of co-cited authors, the centrality of Ernst E, Alimi D and Moraxiitis A was 0.22, 0.14 and 0.10, respectively. This indicates that there is a certain influence of publications by patients in Ernst E, Alimi D and Molassiotis A group, but is not directly influenced by the number of citations.

For journals, a large number of articles published in journals related to cancer, evidence-based medicine, complementary and alternative medicine, pain management and acupuncture. Pain and Symptom Management had the highest impact level. From the trends of journals in the timeline, we found that the Frontier of Oncology was at the forefront of research in the timeline, followed by Pain Medicine, Medicine, BJM Open and Complementary Therapies in Clinical Practice. The top journal in terms of number of articles published is Integrative Cancer Therapies (36 documents), the top journal in terms of number of citations is J Clin Oncol (1106 citations), and the journal with the highest citation centrality is BMJ-Brit Med J (0.3).

For keywords, we found more occurrences of “acupuncture, electroacupuncture, management, pain, fatigue, breast cancer, quality of life, acupressure, prevention,” etc. The highest citation keyword is acupuncture (407 citations) and the highest centrality keyword is alternative medicine (0.29). There are 15 keyword clustering labels based on the clustering map of keywords co-occurrence by log-likelihood ratio calculation. The first cluster are #0 Complementary therapies, #1 Cancer, #2 analgesia, #3 bee venom, #4 breast cancer. And from the clustering labels, we can know that acupuncture acts on cancer pain mostly for the purpose of evaluating the clinical effectiveness of acupuncture therapy for analgesia, and the focus of the study is mostly on the clinical symptoms related to cancer patients that were significantly improved by controlled clinical trials of acupuncture, as well as the differences in the clinical efficacy of the therapy. From the timeline view of keywords, we can infer that the acupuncture for cancer pain academic field emerged in the 1990s or even earlier. There are 16 keywords with the strongest citation bursts from 2000-2022. And among these keywords, “stimulation” lasted for 15 years (2000-2014), “clinical trials” lasted for 13 years, and the last 3 years keywords were “mechanism” and “protocol.”

### 4.2. Research frontiers and trends

In general, we can know the research hotspots and future trends through the keywords or reference co-occurrence, clustering map and keywords emergent words in bibliometrics.

#### 4.2.1. Intervention methods.

Through the keywords co-occurrence and clustering map, we can know that the keyword “electroacupuncture (EA)” may be a major complementary treatment for managing cancer-related pain and improving the lives of cancer patients. For example, EA has been shown to attenuate cancer-Induced bone pain via NF-κB/CXCL12 signaling in midbrain periaqueductal gray.^[15]^ EA reduced inflammatory pain by inhibiting NLRP3 inflammasome activation through CB2 receptors.^[16]^ EA delayed the onset of morphine tolerance in rats with bone cancer pain and downregulated p-PI3K, p-Akt, p-JNK1/2, and β-arrestin2 protein expression in the dorsal horn of the L4-6 spinal cord in rats with bone cancer pain.^[17]^ EA has also been shown to partially alleviate bone cancer pain by inhibiting spinal cord interleukin 1 beta expression in a rat model.^[18]^ EA ameliorated MRMT-1-induced mechanical abnormal pain and thermal nociceptive hyperalgesia in a rat model of bone cancer pain (BCP), and exerted analgesic effects on BCP by reducing P2X3R overexpression and functional activity in the ipsilateral dorsal root ganglia of BCP rats.^[19]^ The above experiments further support EA as a potential treatment option for clinical cancer pain. In addition, EA inhibited chemotherapy induced pains through activation of spinal 5-HT 1A receptors and suppression of p-CaMKII.^[20]^

Furthermore, from the keyword clustering map label #3 (bee venom), we can infer that bee venom acupuncture is also an emerging hotspot in this field. Bee venom is a complex substance produced by honey bees and has now been shown to be effective in a variety of diseases, such as gouty arthritis^[21]^ and epilepsy.^[22]^ Some researches shown that Bee venom acupuncture could regulate action potential thresholds in A-fiber dorsal root ganglion neurons to reduce oxaliplatin-induced neuropathic pain,^[23]^ and central noradrenergic pathway exerts analgesic effects in vincristine-induced peripheral neuropathy in rats by bee venom acupuncture.^[24]^

#### 4.2.2. Types of cancer-related pain.

Based on the co-occurrence graph of keywords and references, we can infer that the common types of cancer pain which are treated with acupuncture including breast cancer pain, joint pain, and neuropathic pain. From the keyword frequency, centrality and clustering, we can infer that acupuncture is centered on breast cancer in the field of treating cancer pain. As of 2023, for women in the U.S., breast cancer, lung cancer and colorectal cancers accounted for 52% of all newly diagnosed cases, and breast cancer alone accounted for 31% of all cancers in women.^[25]^ It is well known that chemotherapy, surgery or medication would lead to a wide range of side effects and affect the quality of life of breast cancer patients. In recent years, some research found that acupuncture could effectively relieve the pain of peripheral neuropathy induced by chemotherapy for breast cancer^[26]^ and musculoskeletal symptoms and arthralgia induced by aromatase inhibitors.^[27]^ These researches provide sufficiently compelling evidence for acupuncture in the treatment of breast cancer-related pain. In addition, acupuncture is one of the important interventions that can help breast cancer patients improve their pain symptoms after surgery.^[28]^ Numerous experts in Evidence-based Clinical Practice Guidelines strongly recommend using acupuncture for the relief of aromatase inhibitor-induced arthralgia in breast cancer patients.

#### 4.2.3. Aromatase inhibitor-associated arthralgia syndrome.

Aromatase inhibitors are the main drugs for adjuvant and metastatic treatment of hormone receptor-positive breast cancer patients. However, the side effects of aromatase inhibitors, mainly arthralgia, seriously affect the therapeutic efficacy of breast cancer or even terminate the treatment, which is also an important reason for poor drug compliance.^[29]^ Based on the keywords clustering map and burst references, some randomized controlled trial (RCT) experiments have shown that acupuncture or electroacupuncture^[30]^ can significantly improve aromatase inhibitor-associated arthralgia^[27]^ and with long-term benefits.^[31]^ These evidences strongly support its effectiveness. At the same time, we found that the popular academic terms labeled in keywords clustering map #9 aromatase inhibitors include acupressure, and acupressure may also be a hotspot for improving or relieving aromatase inhibitor-associated arthralgia in the future. In a pilot study, auricular acupressure was effective in controlling arthralgia in breast cancer survivors.^[32]^ Another research showed that auricular acupressure can modulate inflammatory factors for analgesic purposes.^[33]^ Overall, acupuncture is therapeutically free of significant side effects, but patients’ fear of needling may lead to decreased acceptance and affect treatment effects. Auricular acupressure is a noninvasive and low-cost technique. Compared with acupuncture, auricular acupressure is more acceptable to patients. In the clinic, not only can nurses perform acupressure to enhance the management of aromatase inhibitor-induced arthralgia, but patients can also self-manage their pain anytime and anywhere at home through the guidance of their physicians.

#### 4.2.4. Chemotherapy-induced peripheral neuropathy and neuropathic pain syndrome.

Based on the keywords clustering labels #7 neuropathic pain and #13 chemotherapy-induced peripheral neuropathy, we can infer that acupuncture is one of the frontiers and hotspots in the treatment of post-chemotherapy peripheral neuropathy and neuropathic pain syndromes. Chemotherapy-induced peripheral neuropathy (CIPN) is a common peripheral neuropathy caused by the use of chemotherapeutic drugs such as platinum-based agents, taxanes, vinca alkaloids, and other specific agents, which is characterized by functional impairment and neuropathic pain syndrome.^[34]^ As early as 2006, a case reported corrected 5 cases of pilot regulation of qi and blood in the extremities through an acupuncture protocol, resulting in improvement of symptoms of CIPN.^[35]^ Ting Bao used semi-objective quantitative sensory testing in a RCT and found significant improvements in foot vibration detection thresholds and hand hot/cold detection thresholds after 8 weeks of acupuncture treatment compared with usual care.^[36]^ A RCT in 2018 showed preliminary evidence that acupuncture is effective in reducing the incidence of high-grade CIPN during chemotherapy.^[37]^ A research in 2020 showed significant improvement in CIPN in breast cancer patients during paclitaxel chemotherapy after 8 weeks of acupuncture treatment.^[26]^ Jun J Mao compared acupuncture with usual care in RCT and found that acupuncture significantly improved CIPN symptoms, determined the effectiveness of acupuncture for CIPN.^[38]^

#### 4.2.5. Analgesic mechanisms.

Several reviews of the literature have reported the involvement of various biochemicals such as β-endorphins, IL-1β, dynorphines, substance P in the inhibition of cancer pain by acupuncture or electroacupuncture in animal models of inflammatory pain. Indeed, more exploration of acupuncture for cancer pain is needed to clarify the underlying mechanisms. Based on the burst keywords, we found that “mechanism” is the frontier of current research in the last years. A research has shown that EA could inhibit Toll-Like Receptor 4 signaling and up-regulate Transient Receptor Potential Vallinoid 1 in sensory neurons to attenuate paclitaxel-induced peripheral neuropathic pain in rats.^[39]^ Other research have shown that EA could attenuate cancer-induced bone pain via NF-κB/CXCL12 signaling in midbrain periaqueductal gray,^[15]^ and changes in protein phosphorylation in the dorsal root ganglia of EA-treated BCP (bone cancer pain) rats indicate that mTOR signaling may be a key point in the alleviation of BCP.^[40]^

It is important to notice that the burst of “mechanism” may be related to the landmark contribution of Qiufu Ma’s discovery of research mechanisms associated with acupuncture analgesia,^[41–43]^ which may further bring new insights to researchers with acupuncture for cancer pain.

#### 4.2.6. Protocol for RCTs.

According to the burst keywords, we found that “protocol” can also be used as hotspot and frontier for current research in the last years. Clinical trial protocols of acupuncture for cancer pain have made some recent advances, while there are still some deficiencies. In the systematic review and meta-analysis of clinical evidence on the association of acupuncture and acupressure with improvement of cancer pain, determined the effectiveness of acupuncture and acupressure in relation to improving cancer pain, while the complexity of cancer pain, the variety of treatments and the diversity of needling approaches may be the factors affecting efficacy.^[44]^ A RCT studied the effects of acupuncture on aromatase inhibitor-associated arthralgia in women with early-stage breast cancer,^[27]^ while the study also had several limitations, such as blind method was not applied to patients randomly assigned to the waitlist control group and the primary outcomes were based on relatively short-term measurements, not long-term follow-up. Other researches have shown that acupuncture could significantly reduce pain and uncomfortable symptoms of chemotherapy-induced peripheral neuropathy, while the sample sizes of these studies were limited.^[45,46]^ It should be noted that there are some common deficiencies in the studies of acupuncture for cancer pain, such as small sample size, lack of control group and long-term follow-up. Therefore, more high-quality, large-sample-size, and rigorously designed studies are needed to validate and further improve the clinical trial protocol of acupuncture for cancer pain in the future.

### 4.3. Limitations

There are still some limitations in this study. Firstly, we only used the web of science database to search for related publications, which may exclude some influential papers included from other databases, this might be prone to research bias. Secondly, this study only included articles published in English, which might have overlooked some of the literature.

## 5. Conclusion

This article provides an overview of the research on acupuncture for cancer pain through a bibliometric analysis of annual publication trends, country of publication, authors, research institutions, keywords, and co-citation network citations in the form of knowledge graphs and data visualizations. The literature on acupuncture for cancer pain showed an overall upward trend in the past 20 years. This study can help researchers get a comprehensive picture of the research panorama, historical development, and recent hotspots in acupuncture for cancer pain and provide inspiration for further research, and further exploration of relevant mechanisms is needed to provide a sound scientific basis for the treatment of cancer pain with acupuncture in the future.

## Author contributions

**Conceptualization:** Xia Yang.

**Data curation:** Demin Xue, Jing Liang.

**Methodology:** Xia Yang, Bing Liang.

**Project administration:** Ji Chen.

**Supervision:** Chris Zaslawski, Ji Chen.

**Visualization:** Bing Liang.

**Writing – original draft:** Xia Yang.

**Writing – review & editing:** Xia Yang, Ji Chen.

## Supplementary Material
