# Supplementary material for: Global research trends in acupuncture for cancer pain: A bibliometric analysis
Source: Medicine (Baltimore). 2023 Oct 13;102(41):e34739. doi: 10.1097/MD.0000000000034739 (PMC10578673; doi:10.1097/MD.0000000000034739)
Supplement: Supplementary file 1 [file medi-102-e34739-s001.pdf]

Supplementary Content 1: Top 10 most productive authors.

Rank	Authors	Document	Citations
1	Mao, Jun J.	25	525
2	Bao, Ting	14	401
3	Cohen, Lorenzo	12	490
4	Deng, Gary	9	474
5	Lao, Lixing	9	475
6	Cassileth, Barrie R.	8	466
7	Garcia, M. Kay	7	331
8	Greenlee, Heather	7	867
9	Ben-Arye, Eran	6	81
10	Enblom, Anna	6	63
